# Recent Advances in Dermatitis Herpetiformis

**DOI:** 10.1155/2012/914162

**Published:** 2012-07-02

**Authors:** Kimiko Nakajima

**Affiliations:** Department of Dermatology, Kochi Medical School, Kochi University, Kohasu, Okocho, Nankoku, Kochi 783-8505, Japan

## Abstract

Dermatitis herpetiformis is an autoimmune bullous disease that is associated with gluten sensitivity which typically presents as celiac disease. As both conditions are multifactorial disorders, it is not clear how specific pathogenetic mechanisms may lead to the dysregulation of immune responses in the skin and small bowel, respectively. Recent studies have demonstrated that IgA and antibodies against epidermal transglutaminase 3 play an important role in the pathogenesis of dermatitis herpetiformis. Here, we review recent immunopathological progress in understanding the pathogenesis of dermatitis herpetiformis.

## 1. Introduction

Dermatitis herpetiformis (DH) is an autoimmune blistering, intensely pruritic papulovesicular rash typically located on the elbows, forearms, buttocks, knees, and scalp [1]. The disease can be clearly distinguished from other sub-epidermal blistering disorders by histological and immunological characteristics and presence of gastrointestinal disease. Histopathological findings of the lesional skin of patients with DH are characterized by subepidermal blisters with predominantly neutrophil infiltrates at the tip of the papillary dermis [2]. Direct immunofluorescence (DIF) reveals granular IgA deposition in the papillary dermis [2]. Gluten sensitivity typically presents as celiac disease (CD), a common chronic small intestinal disease. Although DH is highly associated with CD, the gastroenterological symptoms in DH are generally mild or are clinically completely absent [1]. However, inflammatory small bowel changes can often be found by histological examination even in the absence of clinical findings. Both disorders are associated with the IgA class of autoantibodies. A close association between DH and HLA-DQ2 and HLA-DQ8 has been established [2]. Several diseases, including thyroid abnormalities, systemic lupus erythematosus, dermatomyositis, Sjogren syndrome, and rheumatoid arthritis, are associated with DH [3]. As patients with DH have been reported to have an increased risk of intestinal lymphoma, recent reports showed that patients with DH who did not maintain a gluten-free diet had a greater risk for developing lymphoma [4]. On the other hand, several studies have failed to demonstrate an increased incidence of malignant neoplasms in patients with DH [4]. The standard therapy for DH is treatment with dapsone [2]. Here, we highlight the recent immunopathological advances in the pathogenesis of DH.

## 2. *In  Vivo* IgA-Associated Pathogenesis in DH 

The deposition of IgA in the papillary dermis is the immunopathological hallmark of DH. Firstly, it was found that both the perilesional and the uninvolved skin of patients with DH have granular IgA deposition in the papillary dermis [5]. These IgA deposits decreased in intensity or disappeared after the patient maintained a gluten-free diet [3]. Although early studies showed that IgA was associated with bundles of microfibrils and anchoring fibrils below the basal lamina, later studies demonstrated that almost all IgA deposits were related to nonfibrillar components of the skin and other connective tissues [6, 7]. IgA is thought to play an important part in the infiltration of neutrophils into the papillary dermis and in the formation of basement membrane zone vesicles in the lamina lucida. The cutaneous IgA deposits in DH have been shown to function *in vitro* as a ligand for neutrophil migration and attachment [8]. However, the specific IgA antibody responsible for granular deposition in the papillary dermis has not yet been identified definitively.

 Dieterich et al. identified tissue transglutaminase (tTG) as the autoantigen involved in CD [9]. They also showed that circulating autoantibodies to tTG could differentiate patients with DH from those with linear IgA bullous dermatosis [9]. Linear IgA bullous dermatosis often closely mimics the clinical pattern seen in patients with DH [2]. However, the findings of linear IgA deposits at the basement membrane by DIF can distinguish linear IgA bullous dermatosis from DH. Circulating IgA and/or IgG anti-tTG and anti-gliadin antibodies are found in patients with active CD [2]. tTG is a member of the TG family, which in humans consists of nine distinct proteins expressed in a wide variety of cell types [10]. TG family members show conservation, especially of certain enzymatically relevant domains. Strikingly, Sárdy et al. demonstrated that sera from patients with gluten-sensitive disease (GSD) reacted both with tTG and epidermal transglutaminase 3 (TG3) and that sera from patients with DH showed a higher affinity for TG3 [10]. They also demonstrated the colocalization of TG3 with IgA deposition in the papillary dermis of DH patients. In addition, they also revealed that TG3 and IgA complexes at the papillary dermis did not contain tTG. Therefore, they proposed that TG3, rather than tTG, may be the dominant autoantigen in DH. TG3 is homologous to tTG regarding their enzymatically active domains [10]. The function of TG3 in the epidermis involves cross-linking and maintenance of cornified envelop integrity. While TG3 is localized in upper layer keratinocytes, tTG is seen in basal layer keratinocytes in normal skin [10]. On the other hand, TG3 in DH skin is found in the papillary dermis and overlaps with the same sites of IgA deposition. It has been suggested that TG3 might be released from keratinocytes and bound by circulating IgA antibodies in the papillary dermis [10]. Another hypothesis is that preformed circulating complexes of IgA and TG3 might be deposited in the papillary dermis [10]. In fact, these circulating complexes were found in the vessel walls of patients with DH [11]. However, the exact mechanism whereby IgA anti-TG3 deposits are localized in DH skin is not known. 

 Donaldson et al. also reported that patients with DH have TG3 in the papillary dermis overlapping with the deposits of IgA [12]. Additionally, they found TG3 deposits in uninvolved skin at least 5 cm away from the lesions. Moreover, IgA deposits were seen in all skin specimens where TG3 was found, suggesting that TG3 is bound by autoantibodies as the mechanism of deposition. TG3 was not found in the dermis in the absence of IgA. The intensity of IgA by DIF roughly correlated with the intensity of staining for TG3. These findings suggest that factors beyond these complexes are necessary for the formation of DH skin lesions. 

## 3. Granular or Fibrillar IgA Deposits in the Skin of DH Patients

Although DH is most common in Europe and the United States, it is very rare among African Americans and Asians including Japan, perhaps because of differences in the frequency of HLA antigens associated with DH [1]. The incidence of fibrillar patterns of IgA deposits in the papillary dermis of patients with DH has been reported, although it is common that granular deposits of IgA in the papillary dermis are pathogenic for DH [3]. Interestingly, those patients seem to have a decreased frequency of a GSD [3]. In Chinese patients with DH, granular IgA deposits in the papillary dermis were seen in 95.5% (21/22) of patients and fibrillar IgA deposits in the papillary dermis were seen in 1 patient (4.5%) [13]. A recent study also described 3 DH patients with fibrillar patterns of IgA deposition in the papillary dermis and 2 of 3 patients did not have anti-TG antibodies and antiendomysial antibodies [14]. Although patients showing a fibrillar pattern of IgA deposits typically have other clinical findings consistent with DH, it has been suggested that those patients may have a higher incidence of atypical features, such as urticarial or psoriasiform skin lesions, the absence of GSD, or an HLA-B8/DR3/DQ2 haplotype [3]. It is not clear whether this difference in IgA deposits may be associated with the decreased frequency of GSD in patients with DH.

## 4. Immunological Diagnostic Markers of DH

Firstly, Chorzelski et al. reported that IgA antibodies bind to an intermyofibril substance (the endomysium of smooth muscle) in the skin of patients with DH [15]. Amazingly, Sárdy et al. showed that these IgA antibodies have a specificity for TG, particularly epidermal-specific TGs, which were also found in the sera of DH patients as well as CD patients [10]. It is well known now that patients with DH have IgA antibodies that are specific for TG3 and IgA antibodies that react with both TG3 and tTG. A recent study demonstrated that IgA anti-TG3 is more sensitive in detecting DH than any other marker associated with GSD in a large cohort of DH patients [16]. Serum IgA endomysial antibodies (EMAs), which can be detected by indirect immunofluorescence, are serologic markers for both DH and CD. The endomysium is the fine connective tissue sheath surrounding each muscle fiber. Moreover, IgA anti-tTG, which is a major endomysial antigen detectable by enzyme-linked immunosorbent assay (ELISA), has a high range of specificity and sensitivity in DH patients [2]. Levels of anti-tTG and anti-TG3 IgA correlate with the extent of small bowel pathology in CD [2]. Moreover, levels of anti-endomysial, anti-tTG and anti-TG3 antibodies are low in patients with DH and CD that follow a strict gluten-free diet [2]. In addition, serum IgA antibodies directed at gliadin are positive in about 70% of CD and DH patients. However, a potential role for tTG and gliadin IgA antibodies in the pathogenesis of DH has not been proposed. As selective IgA deficiency is about 10 to 15 times more prevalent in patients with CD, no case of selective IgA deficiency in DH has been reported. However, partial IgA deficiency has been reported in DH, indicating that pathogenically directed IgA antibodies were likely sufficient for cutaneous IgA depositions in DH [17]. A recent report indicated that intestinal damage may be associated with the production of IgA anti-tTG and IgA anti-TG3 antibodies in DH patients [18]. Dahlbom et al. demonstrated that high levels of IgA anti-tTG and IgG anti-tTG antibodies are associated with the grade of mucosal villous atrophy and a more severe clinical presentation of CD [19]. However, there are no data available at this time about a possible correlation between serological marker(s) and the clinical severity of DH. 

## 5. Neutrophils in the Pathogenesis of DH

The skin lesions in patients with DH are characterized by the infiltration of neutrophils and IgA deposits in the papillary dermis [2]. When the activity of DH is high, circulating neutrophils in patients with DH show a high level of CD11b [3]. Moreover, neutrophils in skin lesions of DH patients showed increased expression of CD11b, a slightly deceased expression of L-selectin, and increased function of the FcIgA receptor, all of which suggest the partial priming of the neutrophils [3]. IL-8 (CXCL-8) is a chemokine that plays an important role in neutrophil inflammatory responses, including the upregulation of neutrophil expression of CD11b and the shedding of L-selectin, steps that are necessary for firm adhesion to endothelial cells and movement into tissue. It has been previously shown that patients with DH show increased levels of serum IL-8, and IL-8 is also increased in patients who are on gluten-containing diets [3]. A recent study suggested that IL-8 in the sera of patients with DH originates from the small bowel as a mucosal immune response to gluten ingestion [20]. 

## 6. Animal Models of DH

Animal models of gluten sensitivity have been used to better understand the pathogenesis of the disease. Marietta et al. developed a mouse model for DH [21]. They reported an HLA-DQ8 transgenic nonobese diabetic mouse that, when immunized with gluten, develops neutrophilic skin lesions along with cutaneous deposits of IgA. Additionally, the subsequent withdrawal of dietary gluten results in the resolution of the skin lesions. Recently, another excellent model of DH was reported [22]. Zone et al. injected a goat anti-human TG3 antibody (IgG) into recipient immunodeficient (SCID) mice grafted with human skin. Those mice showed papillary dermal immune deposits, and those deposits reacted with both rabbit anti-TG3 and DH sera. Deposition of the transferred IgG appeared in a granular pattern in the papillary dermis of the human skin graft. However, there was minimal neutrophil infiltration. Additionally, the transfer of sera from DH patients resulted in deposits in the papillary dermis, if the sera has a high level of anti-TG3 IgA. Sera with the highest levels of anti-TG3 IgA also had minimal neutrophil infiltration at the basement membrane. In this way, they demonstrated that the passive transfer of an anti-TG3 antibody, both goat IgG and sera from patients with DH, produced granular deposits in the papillary dermis. 

## 7. Conclusion

Advances in genetics and immunology have demonstrated the relevance of the immune pathway to the pathogenesis of DH. Many clinical and experimental studies have established IgA and TG3 as the key players and have provided exciting advances in our understanding of the pathogenesis of DH. Future investigations will further clarify the role of IgA and TG3 and their interplay with other relevant cellular and molecular pathways of the immune systems in DH. 
